# Overexpression of miRNA-145 induces apoptosis and prevents proliferation and migration of MKN-45 gastric cancer cells

**DOI:** 10.17179/excli2020-2777

**Published:** 2020-11-03

**Authors:** Tahereh Zeinali, Leila Karimi, Nayer Hosseinahli, Dariush Shanehbandi, Behzad Mansoori, Ali Mohammadi, Khalil Hajiasgharzadeh, Zohreh Babaloo, Jafar Majidi-Zolbanin, Behzad Baradaran

**Affiliations:** 1 Immunology Research Center, Tabriz University of Medical Sciences, Tabriz, Iran; 2 Gastrointestinal and Liver Diseases Research Center, Guilan University of Medical Sciences, Rasht, Iran; 3 Student Research Committee, Tabriz University of Medical Sciences, Tabriz, Iran; 4 Department of Immunology, Tabriz University of Medical Sciences, Tabriz, Iran

**Keywords:** gastric cancer, microRNA, replacement therapy, apoptosis assay, migration assay

## Abstract

MiR-145 is a tumor suppressor miRNA that its ubiquitously expressed in the body but in numerous types of cancers such as GC, its expression became reduced or sometimes ceased in many subjects. This study aimed at restoring the function of the miR-145 in MKN-45 cells and investigating the function of this miRNA in proliferation, apoptosis, and migration of GC cells. MKN-45 cells were transfected using the PCMV-miR-145 plasmid vector. The MTT, DAPI staining, and wound healing assays were applied to estimate the impacts of ectopic expression of miR-145 *in vitro*. Moreover, alterations in the expression levels of K-Ras, c-Myc, caspase-3, caspase-9, Bax, Bcl-2, and MMP-9 mRNA were measured by qRT-PCR analysis. The findings designated that high expression of miR-145 reduced the proliferation and migration and increased the apoptosis of the MKN-45 cells. These effects occur with concurrent suppression of c-Myc, K-Ras, Bcl-2, and MMP-9 as well as induction of caspase-3, caspase-9, and Bax expression. Exogenous miR-145 influences multiple oncogenic pathways and can be regarded as a promising avenue of future therapeutic interventions for GC therapy.

## Introduction

Gastric cancer (GC) is the third most frequent cause of cancer-associated mortality in the world (Busuttil et al., 2018[4]). This cancer is a disease that has no specific clinical symptoms during its early stages, which causes to the late detection of the disease. Despite the presentation of new diagnostic methods, most GC cases are diagnosed in the advanced and metastatic stages (Tang and Xie, 2013[37]). In this malignancy, the most common places for the metastasis to occur are liver, peritoneum, lung, and bone in 48 %, 32 %, 15 %, and 12 % of metastatic cancer patients, respectively (Riihimäki et al., 2016[32]; Busuttil et al., 2018[4]). Extensive treatments may include chemotherapy, radiation therapy, neo-adjuvant therapy and immunotherapy have been indicated to be helpful in GC therapy. Nevertheless, several reasons such as the poor effect of surgery or cytotoxic effects of therapeutic agents, lack of suitable biomarkers, and occurrence of drug resistance restrict the diagnosis and treatment of GC (Gao, 2013[11]; Sitarz et al., 2018[36]). GC results from a combination of many diverse causes. Among them, *Helicobacter pylori* (*H. pylori*) infection, genetic polymorphisms, and different factors that are related to dietary habits are important causes of GC development. Recent progress in genome science has made it feasible to identify precise molecular mechanisms of GC. Nowadays, microRNA (miRNA) has been recognized to has a pivotal function in the cellular processes by modulating the gene expression and has profound impacts in epigenetic changes in GC (Yasui et al., 2011[42]; Sitarz et al., 2018[36]). The miRNAs are non-coding RNAs, which are about 22 nt in length, usually bind to the target mRNAs in their 3'-untranslated regions (UTR), and restrain their expression by the breakdown of mRNA sequences or inhibiting their translation (Huang et al., 2011[17]; Zhang et al., 2020[47]).

 In numerous recent studies, microRNA-145 (miR-145) was recognized as an anti-tumor miRNA and reduced expression level of this miRNA was observed in several cancer types (Xing et al., 2013[41]; Cui et al., 2014[6]). Growing evidence indicates that this tumor-suppressive miRNA has a pivotal role in a wide variety of tumorigenic properties and regulates several cancer-related properties (Cui et al., 2014[6]). Moreover, numerous studies have revealed that miR-145 can control multiple tumorigenic functions by targeting c-Myc, K-Ras, Bax, Bcl-2, caspase-3, caspase-9, and MMP-9 (Chen et al., 2010[5]; Kent et al., 2010[21]; Qiu et al., 2014[31]; Du et al., 2017[8]; Pan et al., 2018[28]). Also, Yue Zhang et al., conducted a meta-analysis study on miRNAs and found that this miRNA has a significant prognostic value in GC patients (Zhang et al., 2017[49]). Accordingly, it has been indicated that high expression of miR-145 has a suppressing effect on the progression of several types of tumors including the breast, colorectal, lung, liver, melanoma, and pancreatic cancers (Sachdeva et al., 2009[33]; Dynoodt et al., 2013[9]; Yu et al., 2015[45]; Wang et al., 2016[39]; Li et al., 2017[24]; Pan et al., 2018[28]). However, the possible roles and underlying pathways of miR-145 in GC proliferation, apoptosis, and migration are still not well elucidated. Thus, the molecular targeted therapies based on miR-145 expression are of great significance.

In this study, the PCMV-miR-145 plasmid vector was transfected to the MKN-45 cell line. Also, after investigating the expression ratio of the miR-145, we performed *in vitro* tests for indicating the influences of miR-145 restoration on the cell proliferation, apoptosis, and migration of the MKN-45 cell line. Moreover, the expressions of miR-145 target genes including c-Myc, K-Ras, Bax, Bcl-2, caspase-3, caspase-9, and MMP-9 were assessed in the experiments.

## Materials and Methods

### Cell culture 

The gastric adenocarcinoma cell line (MKN-45) was purchased from the Pasteur Institute of Iran. MKN-45 cells were cultured in Roswell Park Memorial Institute (RPMI)-10 % fetal bovine serum (FBS) medium (Gibco Laboratories, Grand Island, NY)) which was supplied with 100 IU/ml penicillin and 100 μg/ml streptomycin. The cultures were preserved at a 37 °C incubator (Memmert, Schwabach, Germany) under a humidified atmosphere with 5 % CO_2_ according to our previous study (Yousefi et al., 2012[44]).

### Amplification of the plasmid vector

The pCMV-miR-145 vector carrying pre-microRNA-145 and empty pCMV as the control group were purchased at OriGene Company (Rockville, MD, USA). Amplification of the plasmid vectors was carried out by molecular cloning in *E. coli* (DH5α) bacteria (Shanehbandi et al., 2013[34]). Resistance to kanamycin was utilized as the selectable marker. After that, plasmid extraction was performed using Maxiprep kit (Yekta Tajhiz, Tehran, Iran). Moreover, quantification of the vectors was carried out using a NanoDrop device (Thermo Scientific, USA).

### Transfection of pCMV-miR-145 in the GC cells

Briefly, MKN-45 cells were cultured at a density of 5×10^5^ per well in 6-well plates and then incubated for 24 h to achieve 40-60 % confluency. Separate wells were considered for pCMV-miR-145-transfected cells as well as the cells transfected with the empty vector. On the following day, the medium was discarded and washed with antibiotics and FBS-free Opti-MEM medium. Six μg of each plasmid vector (OriGene, Rockville, MD) was utilized for transfection of MKN-45 cells using jetPRIME® transfection reagent (PolyPlus, Strasbourg, France). In brief, the plasmid vectors were diluted in 150 mM NaCl to obtain a total volume of 200 μl in a 0.5 ml microtube. In another 0.5 ml microtube, six μl of jetPRIME® reagent was diluted in 194 μl of 150 mM NaCl. Subsequently, the contents of the mentioned reaction tubes were blended and placed for 30 min in dark at 25 °C. The mixture was added to the cultured cells. After 48 h, the cells were monitored to approve successful transfection.

### Evaluation of the transfection efficiency under stable conditions

The pCMV vectors encode a green fluorescent protein (GFP) for monitoring transfection efficiency. Cytation 5 imaging system (Cytation 5, Biotek, Winooski, VT) was employed to detect the GFP-expressing cells. The GFP^+^ cells were evaluated at 475 nm. Then, 48 h later, the cells were examined using flow cytometry. In brief, MKN-45 cells transfected with pCMV-miR-145 or blank vector were suspended in phosphate-buffered saline (PBS) at 1× 10^6^ cells/ml. Stable cells were generated over two weeks of the selection time in the presence of 4 μg/μl Geneticin (G418, Gibco). The stable cells were evaluated by flow cytometry (Miltenyi Biotec, Germany). Concisely, the transfected cells emitted the green fluorescence due to the expression of GFP. Consequently, the portion of the GFP positive cells was evaluated in the FITC channel, and the expression of miR-145 was measured by qRT-PCR.

### Relative quantification of the miR-145 expression in the cells with stable transfection

QRT-PCR was applied to confirm the successful restoration of miR-145 expression in the stable transfected MKN-45 cells. Total RNA was isolated from the cells received Geneticin for two weeks using RiboEx reagent (GeneAll biotechnology, Seoul, Korea). After that, synthesis of cDNA was carried out to obtain miR-145 quantification using universal cDNA synthesis kit (Exiqon, Vedbæk, Denmark). To this end, 10 ng of total RNA was utilized. Following that, qRT-PCR was done in a total volume of 10 μl using 5 μl of 2X SYBR green premix (Takara, RR820L), 4 μl of 1:80 diluted cDNA, and 1 μl of a specific primer for miR-145 (Exiqon, Denmark) on a Light Cycler 96 system (Roche Diagnostics, Mannheim, Germany). The miRNA level was analyzed via the Livak method, in which miR-103 served as a normalizer (Livak and Schmittgen, 2001[25]).

### MTT cell proliferation assay

The capacity of cellular proliferation was assessed with 3-(4,5-dimethylthiazol-2-yl)-2,5-diphenyl tetrazolium bromide) MTT assay. Two weeks after the cell transfection, the cells (approximately 2 × 10^3 ^cells per well) were seeded into 96-well culture plates for four days. The MTT (Sigma, Taufkirchen, Germany) at a concentration of 2 mg/ml was added to the wells and stayed for 4 h at 37 °C. Afterward, 200 µl of dimethyl sulfoxide (DMSO) was added for 30 min at 37 °C to solubilize the crystals. The absorbance was determined with a Sunrise^TM ^microplate reader (Tecan, Switzerland) at 570 nm (Montazami et al., 2015[27]). Each experiment was repeated three times.

### Apoptosis detection by DAPI staining 

4,6-Diamidino-2-phenylindole (DAPI) staining was performed to detect chromatin fragmentation according to previous studies (Karami et al., 2013[19]; Armat et al., 2016[1]). This technique is based on fluorescence creation following the binding of DAPI to DNA molecules. MiR-145 and control cells were cultured in 6-well plates. After 24 h, the cells were fixed using 4 % paraformaldehyde for 15 min and permeabilized using 0.1 % Triton-X-100 for 10 min at RT. DAPI with a final concentration of 1:500 (in PBS) was utilized for staining. Depending on the morphological characteristics, the cells were recognized as normal or apoptotic. Cytation 5 cell imaging system was used to perform imaging.

### Cell migration assay

The cells were seeded at 24-well plates and grown to a confluency of 80 %. Subsequently, a scratch was generated by a sterile yellow pipette tip (10-100 μl). Wound healing cell migration was evaluated by observing the movement of the cells into an acellular zone formed by rubbing the cell lawn by a pipette tip. The migration rate of the miR-145 grafted cells was monitored from 0 h to 96 h by an inverted microscope (Optika, Ponteranica, Italy) and then compared to the migration rate of the control cells.

### Relative quantification of the miR-145 putative targets

Alterations in the expression of K-Ras, c-Myc, caspase-3, caspase-9, Bax, Bcl-2, and MMP-9 genes as putative targets of miR-145 were quantified by qRT-PCR. Three μg of the extracted RNA was used for cDNA synthesis performed by random hexamer primer and RevertAid™ Reverse Transcriptase (RT) (Thermo Fisher Scientific). After qRT-PCR, the expression ratio of the genes was evaluated using the Livak method, and β-actin was employed as a housekeeping gene. Primer sequences for the analyzed genes are presented in supplementary Table 1 (supplementary material). See also the Supplementary data.

### Statistical analysis

The obtained data were analyzed using GraphPad Prism statistical software (Version 6.0, San Diego, CA). All data are shown as the means ± standard deviation (SD). Student's *t*-test and ANOVA were applied to evaluate the statistical significance of the observed differences between the groups. The P values smaller than 0.05 were considered statistically significant.

## Results

### Evaluation of the pCMV vector transfection efficiency

**Figure 1:**
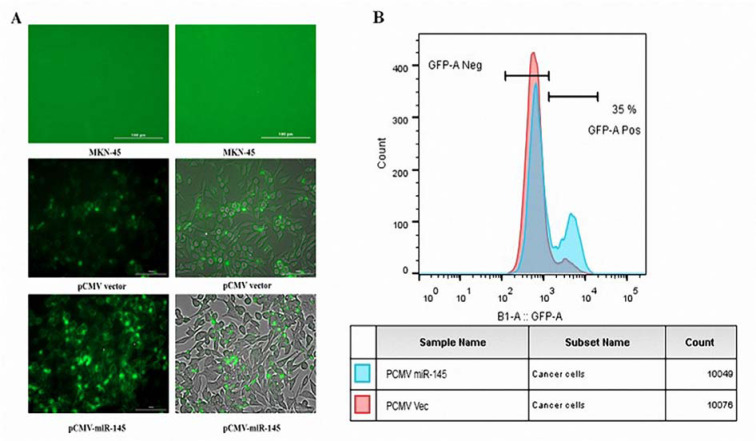
Evaluation of the pCMV-miR-145 transfection efficiency. (A) Green fluorescence was detected in each group of pCMV-miR-145 and pCMV vector-transfected cells, except for the MKN-45 group. The observed green fluorescence was indicative of a successful transfection under Cytation™ 5 system. (B) Flow cytometry was utilized to quantify the expression level of GFP in the transfected cells. The results revealed an increased GFP gene expression in the miR-145 cells.

In order to verify a successful pCMV-miR-145 transfection, the fluorescence phenomenon was detected, and the flow cytometry was performed following the transfection. Cytation 5 system was exploited for the detection of the GFP expression in the transfected cells. The cells expressing a GFP were observed following the transfection by miR-145 (Figure 1A(Fig. 1)). The results of flow cytometry analysis revealed that GFP was highly expresse(Fig. 1)d in the miR-145-transfected MKN-45 cells (Figure 1B(Fig. 1)).

### Quantification of the miR-145 expression level in the transfected MKN-45 cells

**Figure 2:**
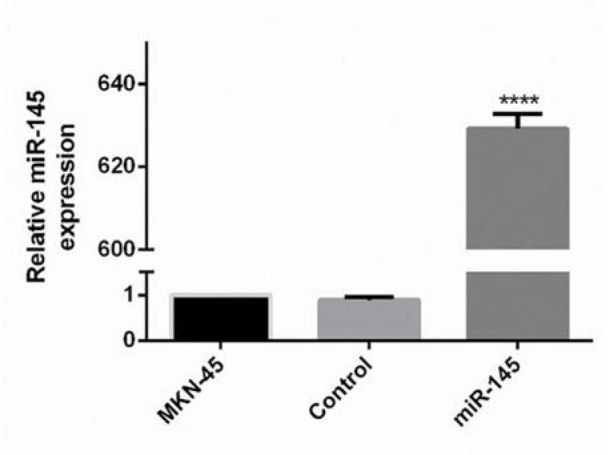
Quantification of the miR-145 expression ratio in the transfected MKN-45 cells. PCR was performed to quantify the expression of miR-145 in each group. QRT-PCR detection results revealed that the expression of miR-145 was statistically promoted in the miR-145 transfected cells. The observed difference between the expression level of the control and transfected cells was statistically significant (**** P<0.0001).

To assess the expression changes of the miR-145 in the miR-145 transfected MKN-45 cells, two weeks after the transfection, qRT-PCR was done. The results designated that the expression of miR-145 was induced 629.2-fold in transfected cells compared to the control group (Figure 2(Fig. 2)).

### Reduction of the cell proliferation by overexpression of miR-145

**Figure 3:**
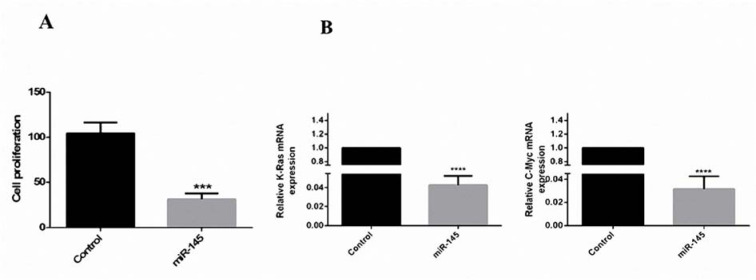
Reduction of cell proliferation by miR-145 *in vitro*. (A) MTT assays revealed that in comparison with the control group, the results of Student's paired t-test were indicative of the reduction of cell proliferation of the miR-145-transfected cells to ~78/7 % (***: p< 0.001). (B) QRT-PCR was done to evaluate the expression ratio of c-Myc and K-Ras. The expression of these genes has decreased 32.25 and 23.8 folds in miR-145-transfected cells, respectively (****, P <0.0001).

The MTT assay showed that in comparison with the control group, the MKN-45 cells, which were transfected with pCMV-miR-145, had a significant growth inhibition rate of 78.7 % (Figure 3A(Fig. 3)). It has indicated that the c-Myc and K-Ras have a crucial effect on the growth control of the cells and their high expression is associated with numerous tumors (Hingorani and Tuveson, 2003[14]; Gabay et al., 2014[10]). Thus, the present study aimed at examining that if miR-145-associated suppression of cell growth is the result of targeting c-Myc and K-Ras oncogenes. As presented in Figure 3B(Fig. 3), the expression of c-Myc and K-Ras were abolished in the miR-145-transfected MKN-45 cells. Thus, miR-145 revealed a statistically meaningful reverse relation with the cellular proliferation of MKN-45.

### Transformation of MKN-45 cells by overexpression of miR-145 following the transfection with pCMV-miR-145

**Figure 4:**
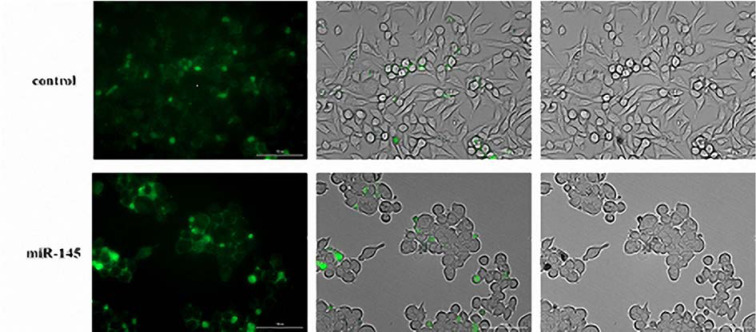
Transformation of MKN-45 cells by miR-145 following the transfection with pCMV-miR-145. The MKN-45 cells containing the empty vector are spindle-shaped or oval. Spindle-shaped cells became rounded cells following the pCMV-miR-145 transfect and miR-145 overexpression.

The pCMV-miR-145 vector was successfully transfected into the MKN-45 cells. MiR-145-transfected MKN-45 cells presented obvious deformations with round cell shape as compared with the cells receiving empty vector (Figure 4(Fig. 4)).

### Induction of apoptosis by overexpression of miR-145

**Figure 5:**
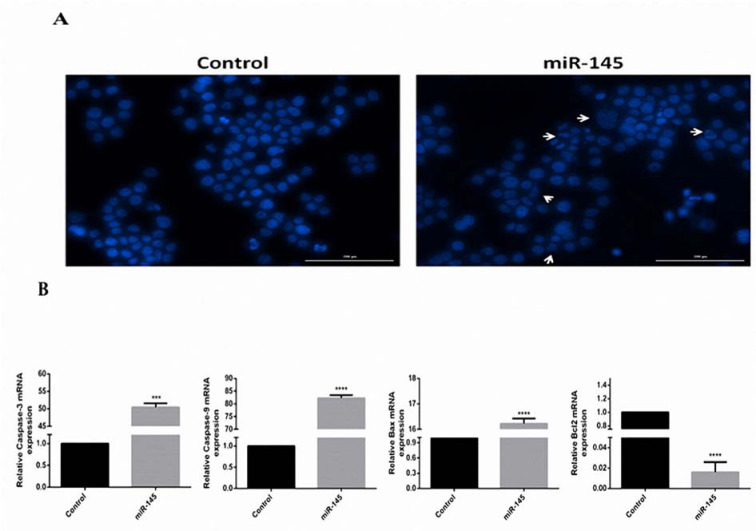
Induction of MKN-45 cells apoptosis by miR-145. (A) DAPI staining was used to perform apoptosis detection in controls and the MKN-45 cells transfected with pCMV-miR-145. The DNA fragmentation was observed under Cytation™ 5 system after DAPI staining. Apoptotic cells are pointed out by arrows. (B). QRT-PCR analysis of the apoptotic genes and β-actin in the control and transfected cells. The expression of caspase-3, caspase-9, and Bax were increased to 50.45, 82.27, and 16.22 folds, respectively. Moreover, the expression level of Bcl-2 was decreased to 66.6 folds.

Apoptosis was assessed by DAPI staining test. MiR-145-transfected cells in comparison with the controls, exhibited increased signs of apoptosis. The fragmented chromatins were regarded as apoptotic nuclei (Figure 5A(Fig. 5)). In addition, miR-145-transfected cells designated the lower expression of the Bcl-2 as well as an increased expression level of the caspase-3, caspase-9, and Bax (Figure 5B(Fig. 5)). Altogether, the mentioned results showed that miR-145 could induce apoptosis of gastric cells.

### Inhibition of cell migration by over-expression of miR-145

**Figure 6:**
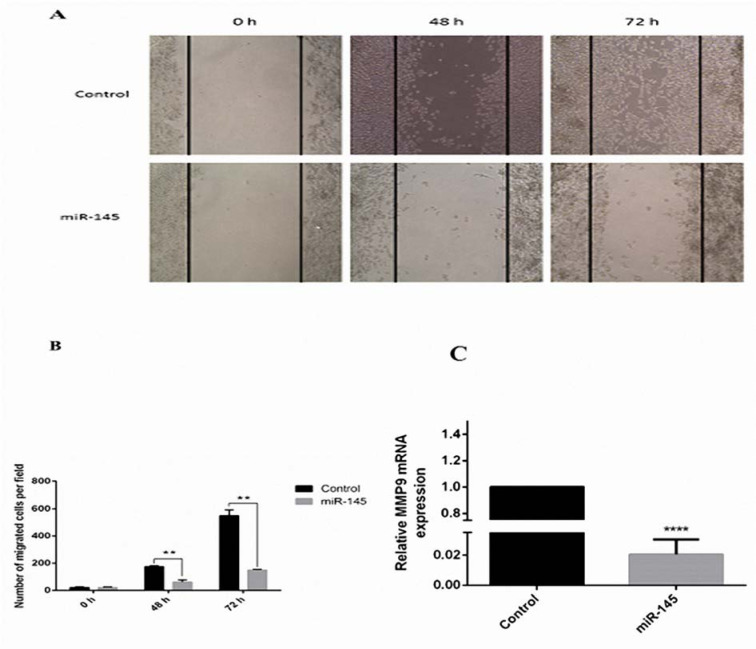
Inhibition of MKN-45 cell migration and decrease of MMP-9 expression level by overexpression of miR 145. (A) The number of migrated cells at the initial point (0 h) and the residual scratch area was photographed by inverted microscope 96 h after wounding. (B) MiR 145 overexpression significantly suppressed the amount of migrated cells in miR-145-transfected MKN-45 cells (magnification, x10) (**P<0.01). (C) High expression of miR 145 decreases the levels of MMP-9 expression up to 50 folds.

The migration test was applied to examine the influences of miR-145 expression on the migratory capacity of the MKN-45 cells. The invasive cell count indicated that the migration capacity was decreased in the miR-145-transfected MKN-45 cells (Figure 6A(Fig. 6)). Additionally, it was shown that the incubation with pCMV-miR-145 decreased the number of closed wounds in comparison to the control group (Figure 6B(Fig. 6)). As MMP-9 is correlated with the metastasis (Deryugina and Quigley, 2006[7]), in this study the expression level of MMP-9 in response to the miR-145 incubation in the MKN-45 cells was evaluated using qRT-PCR analysis. The expression level of MMP-9 was reduced in the miR-145-transfected cells in comparison to non-treated cells (P<0.0001; Figure 6C(Fig. 6)). The mentioned results designated that the elevated level of miR-145 blocks the migration of MKN-45 cells possibly by inhibition of the MMP-9 expression.

## Discussion

**Figure 7:**
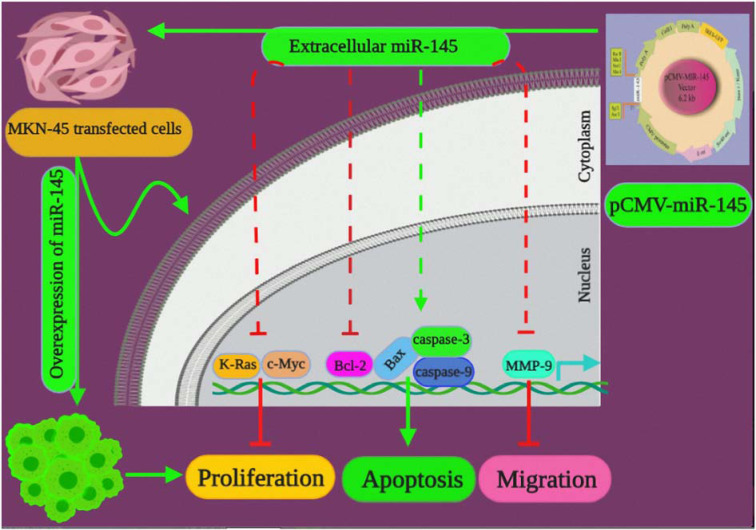
A graphical abstract highlighting the impact of miR-145 overexpression on proliferation, apoptosis, and migration of GC cells. PCMV-miR-145 enhances the gene expression of the miR-145 in transfected MKN-45 cells. Overexpression of miR-145 inhibits c-Myc, K-Ras, MMP-9 and Bcl-2. Overexpression of miR-145 stimulates the caspase-3, caspase-9 as well as, the regulator protein Bax, which regulate apoptosis.

In recent years, a distinct set of miRNAs were recognized to be aberrantly overexpressed in various tumors. In another hand, some other miRNAs that regulate oncogenes were recognized to have reduced expression level that resulted in induced tumor development and progression. Thus, restoring the normal expression level of miRNAs in tumor tissues is a promising therapeutic approach. To reach this goal, delivering miRNAs to the tumor cells through an oligonucleotide mimic or expressing the miRNAs in the cells using a gene vector are feasible approaches. In this regard, the exogenous application of downregulated miRNA in tumor cells may lead to antitumor effects by interfering with several on-cogenic pathways. In addition to this, the results of this approach may cause it becomes more challenging to the tumor cells to initiate escape mechanisms. Another important characteristic of this interference approach is its greatly diminished side-effects. Moreover, the mentioned interference is particularly of a great significance from a therapeutic viewpoint. The main advantage of this approach is the ability of the delivered miRNA in targeting several genes that consequently caused more extensive alterations in protein expression (Bader et al., 2010[3]; Henry et al., 2011[13]; Hosseinahli et al., 2018[15]; Hua et al., 2019[16]; Karimi et al., 2019[20]). Aberrant expression of miR-145 is strictly linked with tumorigenesis and contributes to the pathophysiology of GC (Zheng et al., 2013[50]; Qiu et al., 2014[31]). However, the impacts of miR-145 in GC is not thoroughly recognized. The present study aimed at investigating miR-145 expression in GC and examining its biological function in gastric carcinogenesis. Thus, the tumor-suppressor effects of miR-145 in GC was investigated. Our results revealed that miR-145 restoration in MKN-45 cells by suppressing the proliferation of the cells had important growth-inhibitory influences. Furthermore, miR-145 overexpression induced transformation and apoptosis that indicates its tumor inhibitory effects. Previous studies determined that the ectopic expression of miR-145 led to inhibition of the proliferation of various cancer cells (Chen et al., 2010[5]; Pan et al., 2018[28]). However, numerous findings have designated the importance of miR-145 as a tumor repressing miRNA in GC, its exact underlying mechanisms remain not fully defined. For a more detailed investigation of the tumor inhibitory effects of miR-145 in gastric tumorigenesis, our study evaluated the influences of miR-145 on c-Myc and K-Ras (Figure 7(Fig. 7)). The Myc family oncogenes are deregulated in practically all cancers and are involved in several aspects of the oncogenic process. To date, the direct therapeutic approaches for targeting the Myc activity is not provided, therefore, the essential targets included in Myc deregulation have been used in the treatment of Myc-driven malignancies (Pelengaris and Khan, 2003[30]; Meyer and Penn, 2008[26]; Gabay et al., 2014[10]). Sachdeva et al., indicated that miR-145 was expressed via the phosphoinositide-3 kinase (PI-3K)/Akt and p53 related cascades. This study further revealed that c-Myc was a potential target for miR-145 function. The specific knockdown of c-Myc by miR-145 accounted for the miR-145-related repression of cancer progression in both *in vitro* and animal models (Sachdeva et al., 2009[33]). In line with the obtained results of this experiment in determining the reduced expression of c-Myc due to the miR-145 transfection, a number of other studies addressing various cancer types revealed that miR-145 repressed the expression of c-Myc in various cancers (Chen et al., 2010[5]; Shao et al., 2013[35]; Zhang et al., 2014[48]). The K-Ras gene mutation which is a common event in many malignancies can trigger downstream pathways such as the PI-3K pathway and subsequently can promote cell proliferation and migration (Hingorani and Tuveson, 2003[14]). Meanwhile, our findings indicated that miR-145 dramatically blocked the expression of K-Ras in MKN-45 cells. In line with this finding, another study revealed that K-Ras signaling by stimulating the activity of the ras-responsive element-binding protein 1 (RREB1) caused to inhibition of the miR-143/145 cluster in pancreatic carcinoma. After that, miR-143 and miR-145 targeted K-Ras and RREB1 and established a feedback circuit of Ras signaling (Kent et al., 2010[21]). Similarly, there are inverse correlations among miR-21, K-Ras, and the miR-143/145 cluster in colorectal cancer (CRC) (Yu et al., 2015[45]). Accumulated evidence has also confirmed that c-Myc has a strong tumorigenesis ability that can be increased by cooperation with other oncogenes including Bcl-2, NF-kB, and Ras. Investigation of the collaboration between the c-Myc and Ras or other genes have suggested that the analogous positive feedback loops may have exaggerated oncogenic properties. The evidence reveals that the overexpression of miR-143/145 fully abolishes the transformed phenotype in pancreatic ductal adenocarcinoma cells (Kent et al., 2010[21]; Wang et al., 2011[38]). The mentioned findings are similar to this study, which indicated that the high level of miR-145 in tumor cells led to the transformation of miR-145-transfected MKN-45 cells from spindle-shaped cells to rounded ones and induced cell death (Figure 4(Fig. 4)). Taken together, this study proposed that miR-145 mediates the GC cell proliferation and death by activation of the c-Myc and K-Ras related pathways. In contrast to our finding, some other studies reported the oncogenic function of miR-145. However miR-145 and miR-143 were both downregulated in CRC tissues in comparison with adjacent tissues, it has been indicated that miR-145 promoted the proliferation of metastatic CRC cell line while miR-143 had an inverse function. Other investigations revealed that miR-145-associated oncogenic targets are differently expressed in metastatic and non-metastatic cell line models (Arndt et al., 2009[2]). Some of the mentioned oncogenic genes including Bax, Bcl-2, caspase-3, and caspase-9 contributed to the miR-145-mediated apoptosis. Figure 5(Fig. 5) presents strong evidence revealing that miR-145 overexpression induced apoptosis in miR-145-transfected cells. Also, DAPI staining assay revealed the association of miR-145 and apoptosis. Other findings revealed that upregulation of miR-145 expression reduced the expression of MMP-2, MMP-9, and Bcl-2 and increases the expression of Bax, which leads to the increased Bax/Bcl-2 ratio (Pan et al., 2018[28]). Likewise to our results, numerous studies indicated that miR-145 enhanced the levels of caspase-3 and caspase-9 in NSCLC and glioma cells (Du et al., 2017[8]; Pan et al., 2018[28]). Our results also indicated that miR-145 overexpression could increase the level of caspase-3 and caspase-9 expression in miR-145-transfected MKN-45 cells. Caspase-9 is a key enzyme in the intrinsic apoptotic cascade which is involved in activation of a multiprotein platform and then degradation of the cells into apoptotic bodies. Furthermore, the increased activity of Bax and/or decreased activity of Bcl-2 caused the initiation of apoptosis (Wang et al., 2016[40]; Li et al., 2017[23]). Notably, our study revealed that miR-145 inhibited MKN-45 cell invasion (Figure 7(Fig. 7)). In line with findings of this study, previous studies showed that miR-145-mediated inhibition of metastasis was in part caused by directly targeting SP1, N-cadherin, Ets1, and snail (Jin et al., 2010[18]; Gao et al., 2013[11]; Zheng et al., 2013[50]; Qiu et al., 2014[31]; Ye et al., 2016[43]). Interestingly, MMP-9, an extracellular matrix-degrading enzyme, statistically reduced in miR-145-transfected cells. Nevertheless, the results of luciferase reporter assay indicated that MMP-9 was not a direct target of miR-145. MMP-9 positively correlated with cancer metastasis, and an increased level of MMP-9 expression promotes cancer metastasis (Deryugina and Quigley, 2006[7]; Parmo-Cabañas et al., 2006[29]). MMP-9 activity could be induced by SP1, N-cadherin, Ets1, and snail (Jin et al., 2010[18]; Gao et al., 2013[12]; Zheng et al., 2013[50]; Qiu et al., 2014[31]; Ye et al., 2016[43]; Zeinali et al., 2019[46]). The results of this study suggest that MMP-9 may be a potential target of miR-145. This miRNA may inhibit the MMP-9 expression and then prevents the metastatic activity of the cancer cells. Regarding the importance of the invasive capabilities of the cancerous cells which lead to the reduced prognosis of the patients, miR-145 seems to be an important target for tumor invasion. However, in contrast to our data, Koo et al., recently observed that miR-145 and miR-143, were overexpressed in an invasive glioblastoma cell line, and their reduced expression exhibited a synergistic anti-invasive effect (Koo et al., 2012[22]). The results of Koo et al.,' study and those of our study strengthen the conjectures that miR-145 may have a tissue-specific role in metastasis, as a result of which targeting miR-145 in metastatic cancers needs careful attention.

## Conclusion

Altogether, our findings exhibited that miR-145 is significantly downregulated in GC. To the researchers' best knowledge, for the first time we demonstrated that high expression of miR-145 is able to cause apoptosis and suppresses the malignant phenotype of MKN-45 cells possibly by increasing the expression level of caspase-3, caspase-9, and Bax and decreasing the expression level of Bcl-2, c-Myc, K-Ras, and MMP-9. MiR-145 may thus be proved to serve as a novel target for GC replacement therapy.

## Conflict of interest

The authors have no conflicts of interest to declare.

## Acknowledgement

The authors would like to thank the Immunology Research Center, Tabriz University of Medical Sciences for providing facilities to carry out this research.

## Statement of ethics

All experiments and procedures were conducted in compliance with the ethical principles of Tabriz University of Medical Science, Tabriz, Iran and approved by the regional ethical committee for medical research.

## Funding sources

This study was financially supported by Immunology Research Center, Tabriz University of Medical Sciences, Tabriz, Iran (Grant Number 61694).

## Authors’ contributions

TZ, LK, NH and DS performed the experiments. TZ wrote the article. BM, AM, ZB and JM performed data analysis. KH edited the article. BB designed and supervised the study.

## Supplementary Material

Supplementary material

Supplementary data
