# Supplementary material for: Overexpression of miRNA-145 induces apoptosis and prevents proliferation and migration of MKN-45 gastric cancer cells
Source: EXCLI J. 2020 Nov 3;19:1446–58. doi: 10.17179/excli2020-2777 (PMC7689247; doi:10.17179/excli2020-2777)
Supplement: Supplementary material [file EXCLI-19-1446-s-001.pdf]

EXCLI Journal 2020;19:1446-1458 – ISSN 1611-2156
Received: August 08, 2020, accepted: October 28, 2020, published: November 03, 2020

Supplementary material to:

OVEREXPRESSION OF miRNA-145 INDUCES APOPTOSIS AND PREVENTS PROLIFERATION AND MIGRATION OF MKN-45 GASTRIC CANCER CELLS

Tahereh Zeinali[a,b], Leila Karimi[a], Nayer Hosseinahli[a], Dariush Shanehbandi[a,c], Behzad Mansoori[a,c], Ali Mohammadi[a], Khalil Hajiasgharzadeh[a], Zohreh Babaloo[d], Jafar Majidi-Zolbanin[d], Behzad Baradaran[a,d]*

[a] Immunology Research Center, Tabriz University of Medical Sciences, Tabriz, Iran
[b] Gastrointestinal and Liver Diseases Research Center, Guilan University of Medical Sciences, Rasht, Iran
[c] Student Research Committee, Tabriz University of Medical Sciences, Tabriz, Iran
[d] Department of Immunology, Tabriz University of Medical Sciences, Tabriz, Iran

* **Corresponding author:** Behzad Baradaran, Immunology Research Center, Tabriz University of Medical Sciences, Daneshghah Ave, Tabriz, Iran. Tel: +98 4133371440; Fax: +98 4133371311; Postcode: 5166614766; E-mail address: baradaranb@tbzmed.ac.ir

http://dx.doi.org/10.17179/excli2020-2777

This is an Open Access article distributed under the terms of the Creative Commons Attribution License (http://creativecommons.org/licenses/by/4.0/).

Received: August 08, 2020, accepted: October 28, 2020, published: November 03, 2020

Supplementary Table 1: Specifications of the primers used for qRT-PCR

Genes		Sequence((5' 3')	Product Size
**miR-145**	Target sequence	GUCCAGUUUUCCCAGGAAUCCCU	23bp
**miR-103**	Target sequence	GGCUUCUUUACAGUGCUGCCUUG	23bp
**K-Ras**	Forward	CTCCCTGTGTCAGACTGCTCTTT	154 bp
	Reverse	GGCCTTGCAACCTTGGTCTCTTC	
**C-Myc**	Forward	AGGCTCTCCTTGCAGCTGCT	163 bp
	Reverse	AAGTTCTCCTCCTCGTCGCA	
**Caspase-3**	Forward	GGCGCTCTGGTTTTCGTTAAT	120 bp
	Reverse	CCAGAGTCCATTGATTCGCT	
**Caspase-9**	Forward	GCAGGCTCTGGATCTCGGC	152 bp
	Reverse	GCTGCTTGCCTGTTAGTTCGC	
**Bax**	Forward	TTTGCTTCAGGGTTTCATCCA	151 bp
	Reverse	CTCCATGTTACTGTCCAGTTCGT	
**Bcl-2**	Forward	GAGTTCGGTGGGGTCATGTG	139 bp
	Reverse	CACCTACCCAGCCTCCGTTA	
**MMP-9**	Forward	GGTTCTTCTGCGCTACTGCTG	187 bp
	Reverse	GTCGTAGGGCTGCTGGAAGG	
**β-actin**	Forward	TCCCTGGAGAAGAGCTACG3	131 bp
	Reverse	GTAGTTTCGTGGATGCCACA	

Note: qRT -PCR, quantitative real-time polymerase chain reaction
