# Supplementary material for: Overexpression of miRNA-145 induces apoptosis and prevents proliferation and migration of MKN-45 gastric cancer cells
Source: EXCLI J. 2020 Nov 3;19:1446–58. doi: 10.17179/excli2020-2777 (PMC7689247; doi:10.17179/excli2020-2777)
Supplement: Supplementary data [file EXCLI-19-1446-s-002.pdf]

EXCLI Journal 2020;19:1446-1458 – ISSN 1611-2156
Received: August 08, 2020, accepted: October 28, 2020, published: November 03, 2020

Supplementary data to:

OVEREXPRESSION OF miRNA-145 INDUCES APOPTOSIS AND PREVENTS PROLIFERATION AND MIGRATION OF MKN-45 GASTRIC CANCER CELLS

Tahereh Zeinali[a,b], Leila Karimi[a], Nayer Hosseinahli[a], Dariush Shanehbandi[a,c], Behzad Mansoori[a,c], Ali Mohammadi[a], Khalil Hajiasgharzadeh[a], Zohreh Babaloo[d], Jafar Majidi-Zolbanin[d], Behzad Baradaran[a,d*]

[a] Immunology Research Center, Tabriz University of Medical Sciences, Tabriz, Iran
[b] Gastrointestinal and Liver Diseases Research Center, Guilan University of Medical Sciences, Rasht, Iran
[c] Student Research Committee, Tabriz University of Medical Sciences, Tabriz, Iran
[d] Department of Immunology, Tabriz University of Medical Sciences, Tabriz, Iran

* **Corresponding author:** Behzad Baradaran, Immunology Research Center, Tabriz University of Medical Sciences, Daneshghah Ave, Tabriz, Iran. Tel: +98 4133371440; Fax: +98 4133371311; Postcode: 5166614766; E-mail address: baradaranb@tbzmed.ac.ir

http://dx.doi.org/10.17179/excli2020-2777

This is an Open Access article distributed under the terms of the Creative Commons Attribution License (http://creativecommons.org/licenses/by/4.0/).

EXCLI Journal 2020;19:1446-1458 – ISSN 1611-2156
Received: August 08, 2020, accepted: October 28, 2020, published: November 03, 2020

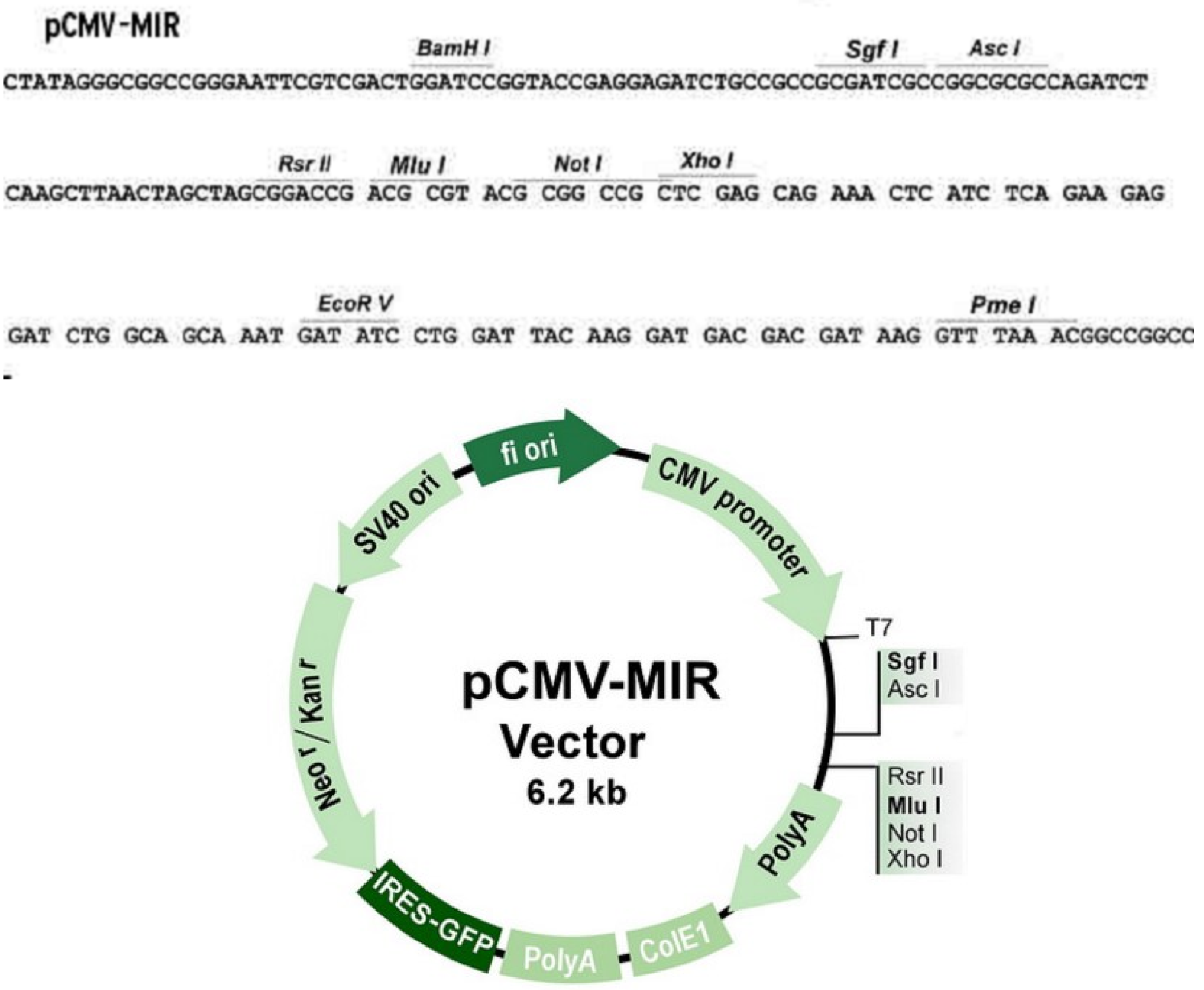

Product image

Vector information links:

https://www.origene.com/catalog/rnai/microrna-expression-plasmids/sc400175/mir145-human-microrna-expression-plasmid-mi0000461

and

https://www.origene.com/catalog/vectors/microrna-vector/pcmvmir/pcmvmir-microrna-expression-vector

MiR-145 sequence

hsa-miR-145-5p :GUCCAGUUUUCCCAGGAAUCCCU

link: http://www.mirbase.org/cgi-bin/mature.pl?acc=MIMAT0000437

Received: August 08, 2020, accepted: October 28, 2020, published: November 03, 2020

Supplementary Table 2: Raw data of MTT assay analysis showing the capacity of cell proliferation of the miR-145-transfected cells in comparison with the control group

5000 cell/well	OD1	OD2	OD3
**Control**	1.49	1.30	1.72
**miR-145**	0.31	0.42	0.51

Note: Figure 3A was extracted from Supplementary Table 2.

EXCLI Journal 2020;19:1446-1458 – ISSN 1611-2156
Received: August 08, 2020, accepted: October 28, 2020, published: November 03, 2020

Supplementary Table 3: Raw data showing investigated genes' expression after transfection of MKN-45 cells with the pCMV-miR-145 or their corresponding control group. The relative expression of each gene was analyzed by comparative threshold cycle (Ct). Ct value was normalized using the formula ΔCt = Ct (investigated genes) - Ct (β-actin). Then formula ΔΔCt = ΔCt (treated) - ΔCt (control) was used. Finally, the formula 2−ΔΔCt was used for estimating relative expression of each gene.

**Group**	**Ct values miR-103**				**Ct values miR-145**				**fold induction**				
	R1	R2	R3	Mean	R1	R2	R3	Mean	R1	R2	R3	Mean	SD
**MKN-45**	20/81	21/08	20/54	20/81	34/37	33/91	34/83	34/37	1	1	1	1	0
**Control**	23/63	24/01	23/25	23/63	38/56	38/34	38/91	38/60333	0/885988	0/855663	0/871671	0/871108	0/01517
**miR-145**	25/72	26/02	25/42	25/72	24/78	24/31	25/12	24/73667	619/5012	632/1247	638/9725	630/1995	9/877368
	**Ct values β-actin**				**Ct values K-Ras**				**fold induction**				
	R1	R2	R3	Mean	R1	R2	R3	Mean	R1	R2	R3	Mean	SD
**Control**	26/58	26/25	26/91	26/58	20/73	21/05	20/41	20/73	1	1	1	1	0
**miR-145**	29/15	28/83	29/47	29/15	32/28	31/32	34/41	32/67	0/042831	0/071236	0/016256	0/043441	0/027495
	**Ct values β-actin**				**Ct values Myc**				**fold induction**				
	R1	R2	R3	Mean	R1	R2	R3	Mean	R1	R2	R3	Mean	SD
**Control**	26/58	27/12	26/04	26/58	37/87	38/13	37/61	37/87	1	1	1	1	0
**miR-145**	22/65	23/93	25/27	23/95	40/46	42/86	46/06	43/12667	0/04303	0/027687	0/023765	0/031494	0/010181
	**Ct values β-actin**				**Ct values Caspase 3**				**fold induction**				
	R1	R2	R3	Mean	R1	R2	R3	Mean	R1	R2	R3	Mean	SD
**Control**	26/58	27/14	26/02	26/58	33/59	33/99	33/19	33/59	1	1	1	1	0
**miR-145**	29/15	28/79	29/44	29/12667	30/48	29/91	31/01	30/46667	51/26847	53/07645	48/50293	50/94928	2/303407
	**Ct values β-actin**				**Ct values Caspase 9**				**fold induction**				
	R1	R2	R3	Mean	R1	R2	R3	Mean	R1	R2	R3	Mean	SD
**Control**	26/58	27/05	26/11	26/58	33/08	32/73	33/43	33/08	1	1	1	1	0
**miR-145**	29/15	28/81	29/49	29/15	29/19	28/82	30/06	29/35667	77/70847	85/62736	83/28588	82/20724	4/068145

EXCLI Journal 2020;19:1446-1458 – ISSN 1611-2156
Received: August 08, 2020, accepted: October 28, 2020, published: November 03, 2020

Supplementary Table 3 (cont.)

**Group**	**Ct values β-actin**				**Ct values Bax**				**fold induction**				
	R1	R2	R3	Mean	R1	R2	R3	Mean	R1	R2	R3	Mean	SD
**Control**	26/58	26/92	26/24	26/58	37/69	38/04	37/34	37/69	1	1	1	1	0
**miR-145**	29/15	28/86	29/44	29/15	36/24	35/88	36/6	36/24	16/22335	17/14838	15/34823	16/23998	0/90019
	**Ct values β-actin**				**Ct values Bcl2**				**fold induction**				
	R1	R2	R3	Mean	R1	R2	R3	Mean	R1	R2	R3	Mean	SD
**Control**	28/19	28/76	28/62	28/52333	26/58	26/14	27/02	26/58	1	1	1	1	0
**miR-145**	28/53	28/26	28/8	28/53	32/45	31/89	32/81	32/38333	0/021642	0/013139	0/020475	0/018419	0/004609
	**Ct values β-actin**				**Ct values MMP9**				**fold induction**				
	R1	R2	R3	Mean	R1	R2	R3	Mean	R1	R2	R3	Mean	SD
**Control**	26/58	26/19	26/97	26/58	25/06	24/67	25/45	25/06	1	1	1	1	0
**miR-145**	29/15	28/91	29/39	29/15	34/21	33/79	34/63	34/21	0/010453	0/011842	0/009227	0/010507	0/001308

Note: Figures 2, 3B, 5B and 6B and C were extracted from Supplementary Table 3.
